# A SynBio community comes of age: Political, academical, industrial, and societal developments in the Netherlands

**DOI:** 10.1016/j.biotno.2022.07.004

**Published:** 2022-08-06

**Authors:** Darshak K. Bhatt, Marjolein E. Crooijmans, Jelmer Coenradij, Alicia Maciá Valero, Maarten Lubbers, Enrique Asin-Garcia, N. Amy Yewdall, Sarah D'Adamo, Nico J. Claassens, Sonja Billerbeck

**Affiliations:** a Department of Medical Microbiology and Infection Prevention, University Medical Center Groningen, University of Groningen, the Netherlands; b Microbial Sciences, Institute of Biology Leiden, Leiden University, Leiden, the Netherlands; c Department of Biochemistry, Groningen Institute of Biomolecular Sciences & Biotechnology, University of Groningen, the Netherlands; d Department of Molecular Microbiology, University of Groningen, Groningen, the Netherlands; e Laboratory of Systems and Synthetic Biology, Wageningen University & Research, Wageningen, the Netherlands; f Institute for Molecules and Materials, Faculty of Science, Radboud University, Nijmegen, the Netherlands; g Bioprocess Engineering, Wageningen University, Wageningen, the Netherlands; h Laboratory of Microbiology, Wageningen University, Wageningen, the Netherlands

**Keywords:** Synthetic biology, Biotechnology, Bioengineering, Community, Science and politics, Science communication, Research landscape, Dutch science funding

## Abstract

Synthetic biology (SynBio) is a rapidly growing scientific discipline. In the Netherlands, various universities and companies are tackling a variety of opportunities and challenges within this field. In this perspective article, we review the current synthetic biology landscape in the Netherlands across academia, industry, politics, and society. Especially within Dutch academia there is an active, though only partially connected, research community involved in various domains of SynBio. Mostly supported by governmental funding, academic research is focusing on top-down synthetic biology, involving the engineering of, for example, bacteria and yeast for bioproduction, as well as bottom-up and cell-free synthetic biology aiming to understand life and build synthetic cells. There is also a large number of talented and motivated students interested in the field, exemplified by the participation and success of Dutch teams in the international iGEM synthetic biology competition. Commercial synthetic biology activities are taking place in various large industrial companies, as well as in start-ups and spin-offs, mostly divided over several ‘SynBio hubs’ in the Netherlands. However, the investment, regulatory and public-perception landscape is not yet optimal to stimulate entrepreneurial activities in SynBio. The Dutch and global society can further benefit from the large promise of SynBio through better integration of people active in the Dutch SynBio field, frequent political and public dialogue, and more attention towards regulatory issues. The recently founded Dutch synthetic biology association SynBioNL aims to contribute to realizing a positive impact on society by stimulating advances of the field in the Netherlands and beyond.

## Abbreviations

**B-Basic**: Bio-Based Sustainable Industrial Chemistry
**BaSyC**: Building a Synthetic Cell
**BBoL**: Building Blocks of Life
**BDC**: Biodesign Challenge
**BE-Basic**: Biotechnology-based Ecologically balanced sustainable industrial consortium
**ENW**: The NWO Domain Science (Dutch: *Exacte en Natuurwetenschappen*)
**IBOS**: Integration of Biosynthesis and Organic Synthesis
**iGEM**: International Genetically Engineered Machine
NWO: Dutch Research Council (Dutch: *Nederlandse Organisatie voor Wetenschappelijk Onderzoek*)
OCW: Dutch Ministry of Education, Culture and Science (Dutch: *Onderwijs, Cultuur en Wetenschappe*n)
**oLife**: origin of Life
**RIVM** the National Institute for Public Health and the Environment (Dutch: *Rijksinstituut voor Volksgezondheid en Milieu*)
**SGW**: The NWO Domain Social Sciences and Humanities (Dutch: *Sociale en Geesteswetenschappen*)
**SynBio**: Synthetic biology
**SynBioNL**: Synthetic Biology association of the Netherlands
**TTO**: Technology Transfer Office
TTW: Applied and Engineering Sciences Domain (Dutch: *Toegepaste en Technische Wetenschappen*)

## 1 Introduction

Synthetic Biology (SynBio) is a field of science focused on (re)configuring biology using engineering design principles, including the improvement of biological systems for applications in the biobased economy.^1^ The field emerged at the beginning of the century and is currently growing into a mature, highly interdisciplinary research field. Innovations in SynBio have contributed to vaccine development,^2^ molecular diagnostics,^3^ cell-based therapeutics,^4^ production of chemicals^5^ and materials,^6^ high-quality animal-free nutrition,^7^ data storage,^8^ and improvements in agricultural production.^9^ Given its potential for a wide range of applications and impact on living systems and societies, SynBio has stimulated discussion around ethics, education, and regulations.^10^ The ongoing dialogue on SynBio is being driven by different entities including academic researchers, government policy makers and advisory bodies, industrial representatives, do-it-yourself synthetic biologists, artists, and the common public.^11^, ^12^, ^13^, ^14^, ^15^, ^16^ To further consolidate the establishment of the SynBio research field and stimulate networks and societal dialogues, in recent years, several SynBio associations have been founded at national^17^, ^18^, ^19^, ^20^, ^21^, ^22^ and international level.^23^, ^24^, ^25^, ^26^, ^27^ Many of these SynBio associations aim to encourage engagement among (young) researchers and stakeholders in the field, as well as to motivate discussions regarding policy and ethics, and promote innovations and (public) education. Many of them have already been successful in reaching out to different levels of society to improve the SynBio discourse.^28^

So far, the Dutch SynBio landscape has grown from an early adoption of practices to a viable and developing research field. However, there is a clear need for an enduring platform that would bring stakeholders together. To this end, SynBioNL (Synthetic Biology association of the Netherlands, https://www.synbionl.com/) has recently been established as a new association coordinated by early-career researchers in the field, to engage researchers, industrial stakeholders, governmental policy makers, risk assessors, students, artists and the public in the Netherlands. As a part of the SynBioNL endeavor, we here discuss our observations of the SynBio landscape in the Netherlands, including funding schemes, the status of SynBio academic research, efforts to recruit new talent via student competitions, the industrial sector, and political engagement, while also providing our vision to create an engaging national community. We performed our analysis through literature review, systematic database searches for publication output and funding, and interviews with stakeholders. We think, given the strong foundations of biotechnology and synthetic biology research and industry in the Netherlands, that SynBio will further flourish in the years to come to increase its impact on society.

## 2 Development and funding of SynBio research in the Netherlands

Due to its diverse applications in solving challenges ranging from the bioproduction of chemicals, to nutrition, data storage and medicine, SynBio has been recognized as a key research field in the European Union.^13^^,^^29^ Also in the Netherlands, SynBio research has obtained numerous funding investments (New and Emerging Science & Technology Pathfinder funding scheme 2004), which has led to large consortia realizing ambitious SynBio ideas (*i.e.,* BaSyC “Building a Synthetic Cell”), and it has harnessed operational industrial applications.^13^ The Dutch Research Council (NWO, Dutch: *Nederlandse Organisatie voor Wetenschappelijk Onderzoek*) serves as the major source of public funding in the Netherlands, including SynBio research. NWO, mainly endorsed by the Dutch Ministry of Education, Culture and Science (OCW, Dutch: *Onderwijs, Cultuur en Wetenschappe*n), allocates nearly €1 billion annually for research (0.125% of GDP in 2020).^30^ This council is as well divided in Social Sciences and Humanities Domain (SGW, Dutch: *Sociale en Geesteswetenschappen*), Applied and Engineering Sciences Domain (TTW, Dutch: *Toegepaste en Technische* *Wetenschappen*) and Science Domain (ENW, Dutch: *Exacte en Natuurwetenschappen*). In order to get an insight into how Synthetic Biology projects are funded in the Netherlands, we accessed the NWO project database. However, due to the interdisciplinary nature of this field, the lack of a closed and consensual definition and the absence of ‘Synthetic Biology’ label in their system, proper analysis and quantification were not possible. Nevertheless, having a decentralized SynBio funding structure can enhance opportunities for acquiring funds as SynBio projects may fall into different divisions (specifically, TTW and ENW).

A substantial amount of SynBio research in the Netherlands is performed within large research consortia and a closer look at them can provide valuable information on long-term SynBio research carried out in the Netherlands over the last 20 years (Supplementary Table 1). Although SynBio is not the main focus within every consortium, their overall research programs use SynBio concepts and methodology to achieve their main goals. Moreover, despite the unavailability of quantitative data, it seems that the synthetic biology community in the Netherlands is growing and there is a substantial number of researchers that describe themselves as working on synthetic biology throughout different institutes and disciplines. Between the different consortia, a distinction can be made between bottom-up and top-down SynBio research. Consortia focusing on bottom-up SynBio such as Building a Synthetic Cell (BaSyC), Building Blocks of Life (BBoL), and origin of Life (oLife) often encompass a similar goal *i.e.,* the construction of artificial (sub)cellular systems towards creating synthetic minimal cells. On the other hand, there are consortia that focus more on top-down SynBio such as Bio-Based Sustainable Industrial Chemistry (B-Basic), Biotechnology-based Ecologically balanced sustainable industrial consortium (BE-Basic), and Integration of Biosynthesis and Organic Synthesis (IBOS). These top-down-focused consortia mostly aim to develop a biobased economy by engineering cellular systems for biosynthesis purposes to produce (fine) chemicals, pharmaceuticals, and bioenergy in a sustainable manner. Together these SynBio consortia encompass a budget of nearly €225 million, publishing more than 300 papers and providing well over 200 PhD and postdoctoral positions in the last 20 years (Supplementary Table 1). Very recently, another large governmental funding was announced involving up to €246 million for the Dutch biotechnology sector, which will include SynBio efforts, under the Bio Booster plan funded by the national ‘growing fund’ (Dutch: *Groeifonds*).^31^^,^^32^

## 3 SynBio research and academia in the Netherlands

In order to observe the impact of research funding on stimulating publications and collaborations within the SynBio research landscape in Dutch academia, we performed a systematic database search based on publication output, author affiliations, and their funding statements. To this end, a search strategy was designed for PubMed to retrieve all studies published till-date that contained terms related to SynBio in their abstract and/or title. The search strategy was thus defined as (((netherlands[Affiliation]) OR (dutch[Affiliation]))) AND (((“biodesign"[tiab] OR “chassis"[tiab] OR “synbio"[tiab] OR “synthetic biology"[tiab] OR “igem"[tiab] OR “biobrick*"[tiab] OR “bio-brick*"[tiab] OR “cell engineering"[tiab]))). 270 articles were found and all literature available in English was included for analysis after manual curation of SynBio-related publications.

Of the 270 articles retrieved from the bibliography analysis, articles with a Dutch affiliation for the first and/or last author were selected. The resulting 189 papers were analysed and sorted into four categories: I) Work supported by national funding, II) Work supported by international funding, understood as funding coming from either EU or another country other than the Netherlands, III) Work supported partially by national funding and partially by international funding and IV) No external funding declared. The funding information retrieved from the articles indicates that over the last twenty years SynBio research has been funded by Dutch (31%), international (21%), or combined Dutch and international funding bodies (31%). This suggests that more than half of the academic output of SynBio research in the Netherlands is, at least partially, funded by national entities. In the last decade, the governmental funding to universities and other research institutes has stagnated somewhat, but the recent government plans aimed to increase this further again [last accessed March 30, 2022].^33^

**Fig. 1:**
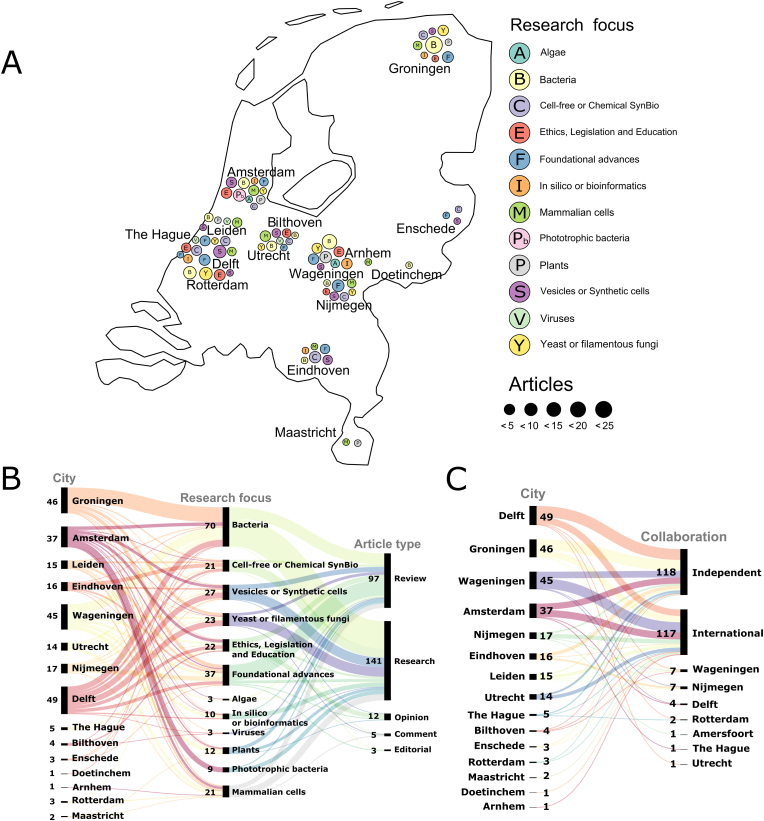
SynBio-based academic research in the Netherlands. **(A)** Distribution of organisms used and research focus on SynBio in the Netherlands. **(B)** Organisms and research focus are discussed in the form of different research publications from different cities. **(C)** Local or international collaboration between academic laboratories in the Netherlands. In **(A)** and **(B)**, projects that engineer or discuss research regarding to algae are labeled as **‘Algae**’; research employing bacteria are labeled as ‘**Bacteria**’; ‘**Cell-free or Chemical SynBio**’ research includes cell-free reactions and protein-networks, in vitro chemical reactions, and enzymatic reactions; ‘**Ethics, Legislation and Education**’ research includes work done in discussing ethics, setting up frameworks for SynBio research and development of SynBio education; ‘**Foundational advances**’ stands for research areas, general or specific, focusing on engineering principles related to all organisms; ‘**In silico or bioinformatics**’ includes computational modeling, software development, and related approaches; ‘**Mammalian cells**’ includes research on engineering mammalian cells; ‘**Phototrophic bacteria**’ includes research on engineering cyanobacteria; ‘**Plants**' includes research on engineering plants; ‘**Vesicles or Synthetic cell**’ research includes the use of vesicles (uni/multi-lamellar), liposomes, microbeads, design and testing of artificial or synthetic cells; ‘**Viruses**’ includes research on engineering viruses; ‘**Yeast or filamentous fungi**’ includes research on engineering yeast or filamentous fungi. In **(B)** and **(C)**, the number of articles is indicated next to the respective labels in black. All data and results are provided in Supplementary Table 2. In **(A)** the map with bubble plots was made using ggplot^70^ in Rstudio. In **(B)** and **(C)** the alluvial diagrams were made using RAWGraphs.^71^

As a result of these funding schemes, SynBio research in the Netherlands has flourished since 2010, as reflected by the increasing number of articles published by Dutch universities (Supplementary Table 2). A diverse repertoire of engineering tools for biological systems has been developed. Analysis of the published articles suggests that so far university-based research labs have mostly focused on engineering bacteria and yeast, as well as developing synthetic cells and cell-free systems, whereas a few labs have also engineered mammalian cells, algae, and plants using principles of SynBio (Fig. 1A). In the future, through funding opportunities and collaborations, we expect that the use of advanced tool kits that are being developed by the global synthetic biology community will facilitate the design of more complex eukaryotic and mammalian organisms.^34^ In reference to the number of published articles, research institutes and universities located in Delft, Wageningen, Groningen, and Amsterdam have made major contributions to the field of SynBio, whereas institutions in Leiden, Utrecht, Eindhoven, and Nijmegen are also actively involved in SynBio research (Fig. 1B). Collaborations between research laboratories could be an important factor in the advancement of SynBio. Interestingly, academic research labs in the Netherlands mostly collaborate internationally on SynBio projects or they work alone, and they less frequently collaborate with other labs in the Netherlands (Fig. 1C). However, we believe there are no specific barriers to internal collaboration, but given the relatively small field researchers often work together internationally. Interestingly, according to our literature review, the frequency of internal as well as international collaboration has increased since 2012 (Supplementary Fig. 1).

## 4 SynBio ethics and politics in the Netherlands

In addition to increased research activities, substantial efforts have been made in discussing politics, ethics, and biosafety policies related to SynBio in the Netherlands. This has been done in the form of research articles, review papers, and opinion pieces, in which especially the Rathenau Institute has played a key facilitating and researching role. In 2011, this institution organised a “Meeting of Young Minds”, where ‘politicians of the future’ – represented by Dutch Political Youth Organisations – had a dialogue on SynBio with ‘scientists of the future’ – Dutch participants from the International Genetically Engineered Machine (iGEM) synthetic biology competition. Following this, the Rathenau Institute has also remained actively involved in bringing SynBio into the debate by reporting on SynBio Politics,^13^^,^^35^ and as a means to invite political parties and non-governmental organisations to actively engage with it in the Netherlands. This was an excellent initiative that provides a platform in the Netherlands to organise an open dialogue between the public, researchers, and policymakers regarding the potential benefits and risks of SynBio in a collaborative manner. Taken together, all these factors leave a promising landscape for novel SynBio research and programs in the Netherlands. However, SynBio has not become a large topic in public and political debates so far.

## 5 iGEM: attracting new talent into the SynBio community

Apart from academic research, international student competitions such as iGEM and BDC (Biodesign Challenge) have played an important role in shaping the SynBio community in the Netherlands. Initiated in 2004, the iGEM competition is an annual, worldwide SynBio event where student teams work to design, build and test their own biological systems during a project of several months. Originally focused on university teams that made contributions to society using SynBio approaches, iGEM has grown into a large community of over 300 teams of university students, high school students, entrepreneurs, and other stakeholders.^16^^,^^36^^,^^37^ However, so far Dutch iGEM teams were mostly from universities. In 2007, the University of Groningen and Delft University of Technology were the first Dutch teams to participate. So far, eight out of the thirteen largest universities in the Netherlands, in addition to the University of Applied Sciences in Rotterdam, have participated in the iGEM competition. Besides regular participation, several Dutch teams have also won the grand prize or received the runner-up award in both over-graduate and undergraduate categories (Table 1), as well as numerous other awards. Winning projects focused on various challenges, such as meat spoilage,^38^ a diagnostic device for antibiotic resistant bacteria in cattle^39^ and a diagnostic device for pathogens.^40^^,^^41^ The relatively high number of participating and well-ranked teams (given the size of the country) highlights the motivation of talented students for SynBio, as well as the generally good institutional support for iGEM and SynBio research by various research groups and universities.

**Table 1:** Dutch teams in the finals of the iGEM competition.

Year	Team	Project	Final ranking
2012	Groningen University (Undergrad)	*The Food Warden*: an alternative method of assessing edibility by using an engineered strain of *Bacillus subtilis* to detect and report volatiles in spoiling meat.	#1
2014	Wageningen University & Research (Overgrad)	*BananaGuard*: engineering the native soil bacterium *Pseudomonas putida* to protect banana plants against *Fusarium oxysporum* infection.	#2
2015	Delft University of Technology (Overgrad)	*3D micro(be) printing*: development of a new *Escherichia coli* system and printer for DIY-biofilm production in a reproducible and automated way.	#1
2016	Wageningen University & Research (Overgrad)	*Bee-T* Saving honey bees from *Varroa destructor*: arming bacteria with targeted and specific toxin production against mites.	#2
2017	Delft University of Technology (Overgrad)	*CASE13A - Cutting our way through the antibiotic resistance problem*: a system for the detection of antibiotic resistance genes in agricultural pathogens.	#1
2019	Wageningen University & Research (Overgrad)	*Xylencer - silencing Xylella fastidiosa*: development of an effective solution for plant-disease by using bacteriophage therapy.	#2
2020	Leiden University (Overgrad)	*Rapidemic*: a novel modular point-of-care diagnostic tool for rapid epidemic response.	#1
2021	Delft University of Technology (Overgrad)	*AptaVita*: a novel and modular aptamer-based rapid diagnostic test for vitamin deficiencies.	#2

In the Netherlands, iGEM teams have collaborated closely with the RIVM, the National Institute for Public Health and the Environment. According to the RIVM, their close collaboration with iGEM teams enables them to “gain insight into topical developments in biotechnology and the ways in which safety needs to be taken into account”.^42^ For example, in 2018, the RIVM developed the *Safe-by-Design Serious Game*, challenging iGEM teams to develop a safe-by-design product that would in the end be market ready.^43^

Besides the fact that most Dutch universities offer credits for participating in the iGEM competition, a variety of SynBio related Bachelor and Master programs covering key aspects of SynBio are also offered. Examples include Biotechnology (Wageningen University), Molecular Genetics and Biotechnology (Leiden University), Nanobiology (Delft University of Technology), and Biomolecular Sciences (University of Groningen). Courses fully focused on SynBio include About Building Cells (Wageningen University), Systems and Synthetic Biology (Wageningen University), Engineering of Living Systems (Delft University of Technology), and Synthetic Biology (Maastricht University). Simultaneously, both academics and the industry offer SynBio thesis and internship projects to both Bachelor and Master students.

## 6 SynBio and the art community in the Netherlands

Since 2016, Biodesign challenge^15^ has launched an annual competition that bridges art, design, and biotech to encourage students to explore biotech's entanglements within society. Delft University of Technology (2019), Amsterdam University of Applied Sciences (2021–22), Design Academy Eindhoven (2021–22), and HKU University of the Arts Utrecht (2022) have all participated in the BDC with biodesign projects while considering the broader implications of emerging biotech. The increasing participation of Dutch institutes in SynBio projects related to art and design is also paralleled by the interest of the contemporary artistic community in the Netherlands (*e.g.*, portraits made by Phelim Hoey using photographic *Escherichia coli*, 2014–2015^44^^,^^45^). Of note, the Waag institute in Amsterdam has created an open wet-lab space intending to promote bioart and do-it-together/do-it-yourself biology in the Netherlands. As a part of the European S + T + ARTS network, its ‘Art science’ project facilitates artist-in-residences, artistic research, and collaboration between scientists and artists. In this regard, MU Hybrid Art House in Eindhoven has also promoted art inspired by science and SynBio, by being a platform that encourages art lovers in the Netherlands and provides a mix of design, pop culture, and new media.^46^, ^47^, ^48^

## 7 SynBio innovations and industry in the Netherlands

SynBio innovations in academia generate knowledge that can be capitalized by established enterprises, and moreover, they have the potential to stimulate the economy by creating new companies built around these innovations. Interestingly, SynBio engineering concepts (*i.e.,* the design-build-test-learn cycle) already play an integral part in many large companies and start-ups in the Netherlands that use the power of nature to their advantage. For this section, views about the synthetic biology industrial landscape of the Netherlands were collected by conducting exploratory interviews to start-up representatives. Conversations were semi-structured consisting of open-ended and general questions to prevent bias and to allow in-depth discussions. These interviews were not intended as a source of information for a formal analysis. Instead, their purpose was purely informative and supportive, and therefore subjective.

We found that the Dutch SynBio industry generally falls into two categories. The first category consists of larger chemistry or pharma companies that were established before the rise of this field. These companies have found recent interest in expanding their R&D into SynBio approaches.^9^^,^^49^, ^50^, ^51^ The other category consists of companies that are spin-offs from research done at universities across the country. Many of these initiatives are still in the early phase and therefore strongly connected to the university where the innovation originally was discovered. Each university in the Netherlands has a Technology Transfer Office (TTO), departments or independent non-profit organisations affiliated to their respective universities with the goal to facilitate the commercialization of research and knowledge being generated at the universities.^52^ TTOs provide legal and financial aid but not under a unified strategy, given that each TTO office operates independently and is guided only by their local culture.

All Dutch universities have their own independent TTO, which results in a variety of strategies. This applies mostly to the way in which the different universities have anchored the activities in the area of knowledge promotion in the organizational structure. The first option would be one in which the promotion is entrusted to a centre of expertise. In the second, this responsibility is entrusted to faculties or institutes, instead of relying on a central organization. The third option would be the outsourcing of the knowledge promotion to an intermediary. The majority of the universities now opt for a self-contained organization, typically in the shape of a holding private company, fully owned by the university, which has been proven to mediate the target activities in a faster, cheaper and more effective manner. Out of the fourteen main universities of the Netherlands, twelve have now one (some even two) of this kind of companies, housing spin-offs and promoting knowledge to innovation.

TTOs at different universities also vary in the rules and procedures for patent expenditure and income. A number of them use the proceeds of the patents for common patent funds, to file new patents, while others “reward the investors” by investing in new research. Other aspects specific to each TTO are the support for newcomers, and the availability of incubators, the degree of contact with the business world, and the offer of education related to entrepreneurship.

One consequence of this type of support system and the relatively low level of national collaboration between universities (Fig. 1C) is the creation of decentralized knowledge hubs. In the Netherlands, the SynBio industry is divided into regions and districts related to those specific university specialties and knowledge. The science parks surrounding these universities have higher activities in start-ups and companies related to the expertise. These regionally divided SynBio activities could benefit from more integrated activities and collaborations across universities. Recently strengthened alliances across different universities (*e.g.,* 4TU Alliance of Technical Universities in Delft, Twente, Eindhoven and Wageningen, Leiden-Delft-Rotterdam alliance, and the Centre for Living Technologies^53^ supported by Eindhoven University of Technology, Wageningen University & Research, Utrecht University and University Medical Centre Utrecht) could potentially foster this.

Additionally, many cities have specialized incubation and acceleration programs supporting the development of new ventures in their area of expertise (*e.g.,* YES!Delft,^54^ Starthub Wageningen,^55^ PLNT Leiden^56^). For example, the Leiden Bioscience Park has an incubator named *Unlock_*.^57^ Violette Defourt is the CEO and co-founder of Rapidemic^58^ (product of the 2020 Leiden iGEM project), a company that aims to make quality healthcare accessible to everyone. Her start-up is participating in one of these programs and describes her experience as: “These programs are a game changer! *Unlock_* has many partners that facilitate lab space and a social environment to meet other life science start-ups. They facilitate you with everything you need to grow”.

There are several funding opportunities for early-stage start-ups in the Netherlands to transition from research to company. Most interesting are the government-supported grants (NWO Take-off^59^ phase 1 and NWO Take-off phase 2) or loans that are in place to stimulate the use of innovative research for industrial applications. However, these budgets (of €40 K to €250 K) are relatively small for biology-related projects that require more time for research and development, expensive equipment, labs, and expensive consumables compared to other more established companies. These budgets only provide the first initial boost to reach out to bigger private and public funding opportunities. Proof-of-concept phase investing funds such as UNIIQ^60^ (€28.8 M) are partly funded by the European Union and provide close connections between TU Delft, Leiden University, and Erasmus Medical Center. However, these opportunities are limited to entrepreneurs within the province South Holland. Possibly the recently awarded BiotechBooster (*Groeifonds*) can alleviate some of these funding limitations. According to Niek Savelkoul, CEO and co-founder of Scope Biosciences^61^: “Funding for these kinds of projects is limited. Grants of a maximum of €250 K are inadequate amounts of money to do this type of research and there are no subsequent options when that money runs out. Perhaps venture capital is a good alternative, but getting these investors interested in this field took a lot of time. Getting people excited about these types of technologies in, for example, the United States is much easier”. Luckily, the landscape is changing now as companies abroad are looking into expansion in the European market. “Eventually, many of these companies that started in the US need the market that is present in Europe” according to Prof. dr. Peter Punt, co-founder of Utrecht-based Dutch DNA, which was acquired by the Boston-based company Ginkgo Bioworks in 2021.^62^

However, starting a business in a relatively young field is not easy. The blurry distinction with the overarching biotechnology field makes it difficult to envision SynBio not as an approach but as a field itself. These circumstances are not detrimental but can nonetheless have negative consequences at a regional level. Within the field of biotechnology, there are many companies, research laboratories, regulatory bodies, and associations at every level that constitute a great biotechnological ecosystem. However, their goals, methodologies, interests, and applications are too diverse for an optimal exploitation of the network, preventing many of these institutions from feeling part of an actual community. We discussed this matter with several representatives from Dutch start-ups and, and while they acknowledge their link to SynBio, they see themselves more as biotech-oriented companies. The fact that what many consider a good representative of a SynBio company in the Netherlands (*i.e.,* EV Biotech) includes in its name the word “Biotech” may showcase two things. On the one hand, it could result from the aforementioned hazy differentiation between SynBio and Biotech. On the other hand, it could also be an indicator of the safe distance companies position themselves to avoid a negative perception associated with the term SynBio. Society, and most likely the institutions too, prefer the term “biotechnology” over “synthetic biology”.^63^ While the former has become more popular among the non-specialized public, the latter remains more unknown and evokes more explicitly that intimidating feeling of “artificialness”.

Dutch policy, like most European administrations, considers biotechnological matters from a precautionary perspective and a culture of compliance. Entrepreneurship is intrinsically linked to risk-taking, but the current regime complicates the process even further. We observe a lack of a large entrepreneurial atmosphere concerning SynBio in the Netherlands, probably derived from the high stakes attached to this technology precisely as a consequence of the aforementioned regulation and public perception. Contrastingly, some positive notes in terms of entrepreneurship can be highlighted from the Dutch education system, namely its highly applied and international character, both powerful attributes to launch entrepreneurial projects. We notice that executive boards and founders of start-ups in the Netherlands often include members from other countries. We hypothesize that the proficiency in the English language of the country in general, but particularly in the highest levels of companies and institutions, has attracted and facilitated the establishment of enterprising talent from abroad. Additionally, the Dutch are striving to stimulate equality by supporting women on executive boards and funding female-only driven companies,^64^ although more efforts seem to be necessary to stimulate gender and other types of diversity.^65^ There is still a lot of room for synthetic biology companies in the Netherlands but having an idea and creating a start-up in the Dutch culture at the university remains very difficult. “Europeans are incredibly risk-averse, and in the Dutch culture, people are sometimes hesitant to celebrate others' successes” according to Linda Dijkshoorn, CEO and founder of EV Biotech.^66^

## 8 Conclusion: SynBioNL – the platform for connecting all stakeholders

While Dutch academia and industry have begun to value SynBio^67^ and while the success of Dutch iGEM teams showcases the motivation and talent of young researchers who want to enter the field, SynBio efforts feel disconnected on many levels within the community, if we can even speak of one. According to Federico Muffato, CEO and founder of Digi.Bio: “If you look at many different verticals in the Netherlands, there is no interconnection”. Despite empowering the scientific endeavors within Dutch SynBio, funding schemes such as research consortia have a limited lifetime (5–10 years), and there have been recent calls^67^ to develop more long-term, multi-stakeholder ecosystems that involve universities, industries, and society. Fortunately, these issues that the young Dutch SynBio ecosystem faces can be resolved by a centralised connector that facilitates the needs of each stakeholder present in the field. We believe that the network within the Dutch SynBio community could be stronger.

We were inspired to establish SynBioNL by learning from the success of GASB (German association for Synthetic Biology) in promoting connections, research and events related to SynBio in Germany.^17^ Additionally, it is important to point out that funding, educational and political frameworks regarding SynBio are organized at a national level, so it is crucial that the Netherlands has a SynBio association of its own. By filling this gap, SynBioNL will be active in steering dialogue between local laboratories, industry, and policymakers in addition to collaborating with other EU and international SynBio communities. We believe that the role of SynBioNL would be crucial in this process as associations from other countries will not have the experience and compatibility to solve issues or raise opportunities related to SynBio in the Netherlands. However, SynBioNL will also learn from the lessons of other SynBio associations for optimal results. In this regard, we envision SynBioNL, an association built by PhDs and Postdocs for the wider SynBio community to connect diverse stakeholders. The SynBio landscape in the Netherlands already contains active stakeholders (*e.g.,* Rathenau instituut, HollandBio, Young NBV) carrying out activities that can potentially synergise with SynBioNL to create adaptive funding frameworks, in expediting the establishment of safety and research guidelines by working with RIVM, and in promoting educational discourse (*e.g.,* iGEM and BDC) in the Netherlands. The aim of such an ecosystem would therefore not only be restricted to gathering funding or promoting research but also to encouraging imaginative narratives. For example, discussing the plausible futures for synthetic cell applications^67^ will not only assist the acceptance of this technology within civil society, easing distrust, but will also enrich SynBio by including diverse perspectives that align with the values of a multifaceted Dutch society.^13^^,^^68^^,^^69^ Moreover, we believe that there is potential to cultivate collaborations between local universities and industries in the Netherlands, for example, where biological system diversity can be improved to provide alternatives to common bacterial- and yeast-based designs. Civil society needs to be engaged in the distinctions and differences of SynBio applications. With a more knowledgeable and engaged public, opinions may soften or a middle ground can be reached to create viable futures for the many possible synthetic biology applications.

## Author contributions

Conceptualization: DKB, MEC, JC, AMV, ML, EAG, NAY; Data curation: DKB, MEC, JC, AMV, ML, EAG, NAY; Formal analysis: DKB, MEC, JC, AMV, ML, EAG, NAY; Investigation: DKB, MEC, JC, AMV, ML, EAG, NAY; Methodology: DKB, MEC, JC, AMV, ML, EAG, NAY; Supervision: SD, NJC, SB; Validation: SD, NJC, SB; Visualization: DKB; Writing - original draft: DKB, MEC, JC, AMV, ML, EAG, NAY; Writing - review & editing: DKB, MEC, JC, AMV, ML, EAG, NAY, SD, NJC, SB.

## Declaration of competing interest

The authors have no conflicts of interest to disclose.

## Appendix A Supplementary data

Multimedia component 3 Multimedia component 3

figs1 figs1
