# Supplementary material for: A SynBio community comes of age: Political, academical, industrial, and societal developments in the Netherlands
Source: Biotechnol Notes. 2022 Aug 6;3:62–9. doi: 10.1016/j.biotno.2022.07.004 (PMC11446357; doi:10.1016/j.biotno.2022.07.004)
Supplement: Multimedia component 1 [file mmc1.docx]

**Statement of Consent**

for participation in scientific research of SynBioNL

Research goal: To elucidate the state of the synthetic biology landscape in The Netherlands using as source the opinions and experiences of relevant stakeholders of the national field.

- I know what the research is about. I have had the opportunity to ask questions.
- I consent to participating in the **interview**.
- I know the interview is recorded and transcribed. My contribution may be used for a scientific publication.
- My contribution (voice, image and transcript record) is handled **confidentially**, but fragments of my answers can be **quoted** within the manuscript with my name.
- Without my consent my quotes will not be used in the research. I know that I will be offered to read them in the context of the manuscript before publication and will have 5 working days to express my opinion about the utilised quotes.
- My contribution (voice, image and transcript record) will not be shared with others, except the authors of the article.
- My contribution (voice, image and transcript record) will be saved on a secure network.
- My contribution (voice, image and transcript record) will be saved to maximally 5 years after the initial research. Afterwards, it will be deleted.
- I know I can refuse to answer research questions. I do not need to indicate why the question is not answered.

**Name: ……………………………………………………………………………………………....…**

**Signature: …………………………………… Date:……………………**

**Description of research**

This interview is aimed to elucidate the state of the synthetic biology landscape in The Netherlands using as source the opinions and experiences of relevant stakeholders of the national field. Quotes obtained from the interviews will be used to support arguments and guide the storyline of a manuscript intended for publication in a peer-reviewed scientific journal. This publication is one of the first works and sets the stage for SynBioNL, the Synthetic Biology Association of The Netherlands.

**Contact details**

Researchers: **Marjolein Crooijmans,** [**m.e.crooijmans@biology.leidenuniv.nl**](mailto:m.e.crooijmans@biology.leidenuniv.nl)**; Enrique Asin-Garcia,** [**enrique.asingarcia@wur.nl**](mailto:enrique.asingarcia@wur.nl)

Data Protection Officer of Wageningen University: [functionarisgegevensbescherming@wur.nl](mailto:functionarisgegevensbescherming@wur.nl)

Please see <https://www.wur.nl/en/About-Wageningen/Integrity-and-privacy.htm> for more information concerning your rights when it comes to your contribution.
