# Supplementary figures and images for: A SynBio community comes of age: Political, academical, industrial, and societal developments in the Netherlands
Source: Biotechnol Notes. 2022 Aug 6;3:62–9. doi: 10.1016/j.biotno.2022.07.004 (PMC11446357; doi:10.1016/j.biotno.2022.07.004)

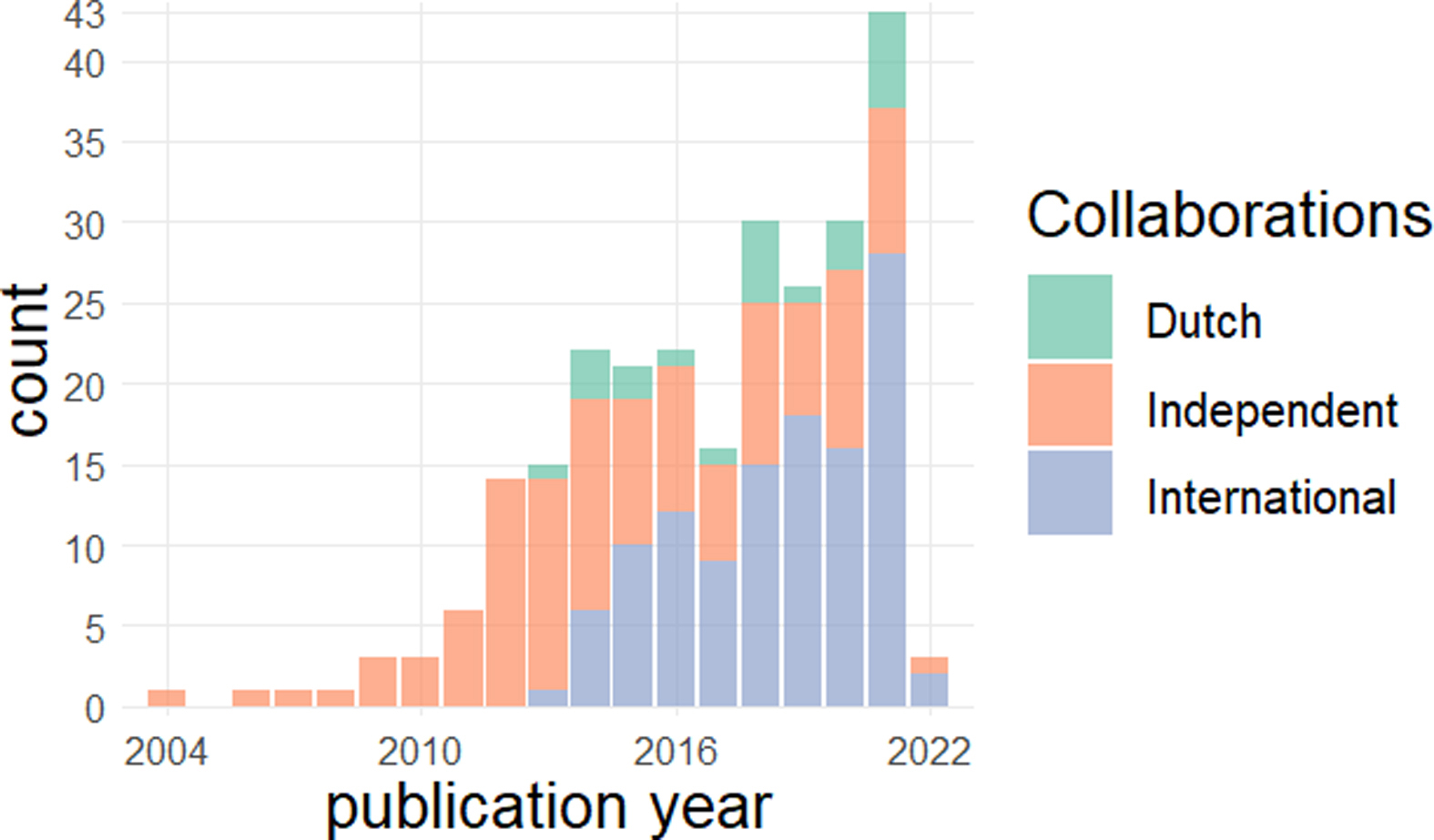

Supplement: figs1 [file mmcfigs1.jpg]
